# Commentary: Postoperative hypothalamic-pituitary dysfunction and long-term hormone replacement in patients with childhood-onset craniopharyngioma

**DOI:** 10.3389/fendo.2024.1371424

**Published:** 2024-02-27

**Authors:** Lukas Andereggen, Emanuel Christ

**Affiliations:** ^1^ Department of Neurosurgery, Kantonsspital Aarau, Aarau, Switzerland; ^2^ Faculty of Medicine, University of Bern, Bern, Switzerland; ^3^ Department of Endocrinology, Diabetology and Metabolism, University Hospital of Basel, Basel, Switzerland

**Keywords:** craniopharyngioma, hormone replacement therapy, obesity, hypothalamic involvement, recurrence, neuropsychological health

We read with great interest the article by Miao et al. (1) reporting the long-term results of patients who underwent surgery for childhood-onset (CO) craniopharyngioma (CP). The number of patients included in the study is impressive, with 200 CP patients having CO. However, long-term follow-up data (median 29.7 months, range 19.0 to 40.3) was available for only about one third (71 patients, 36%), and among them, only 62 patients (31%) had documented regular visits at the endocrine department. The study recorded significant endocrinological sequelae post-surgery, with 94% of the 62 patients having undergone at least one form of hormone replacement therapy during the noted long-term follow-up. Miao et al. eloquently detailed the treatment plan for patients with endocrine dysfunction, emphasizing the crucial role of monitoring by an experienced pediatric endocrinologist. In addition to detailing the subsequent hormonal replacement therapy and the recorded tumor recurrence rate in 18 (26%) patients, it is crucial to incorporate long-term strategies into the adjuvant treatment plan in this patient cohort. Namely, in a comprehensive 10-year follow-up study of CP patients, we observed significant occurrences of hypothalamic obesity (HO) and enduring endocrine deficiencies (2). In addition to ongoing monitoring of endocrine deficiencies, it is imperative to implement early weight control programs and maintain continuous multidisciplinary care for this patient cohort (2).

As suffering from CP is currently a chronic, lifelong condition, the incorporation of long-term follow-up data as an outcome measure is essential to ensure and validate the therapy’s long-term safety and efficacy. It serves as a vital control mechanism for evaluating and adjusting the provided medical treatment (3). In particular, being a chronic disease (4), it highlights the necessity of conducting extended follow-up studies on the outcomes of surgical interventions to understand the sequelae experienced by individuals with CP over time. Namely, beside to the potential lifelong morbidity caused by the tendency of CP for local recurrence (5), it’s important to note risk factors that affect resection rates (i.e. prior radiation, tumor size) (6–8), recurrences (i.e. extent of resection, tumor size), and the quality of life (QoL, i.e. tumor size and entity, surgical approach, vision impairment, endocrine dysfunction, hypothalamic involvement) (9–12) during a longitudinal follow-up period. Regarding the likelihood of recurrence following transsphenoidal compared to transcranial approach, it would be intriguing to delve into more detailed information, such as the extent of tumor resection or the initial size of the tumor (1).

In terms of the specified hormonal replacement therapy, the use of recombinant growth hormone (rGH) therapy in CP patients has been found to be safe, with no reported association with the recurrence or progression of pituitary adenoma or benign tumors (13). Although traditionally a waiting period of at least one year for stable disease is recommended for malignant lesions, recent evidences suggest that this interval could be as short as three months for children with radiologically confirmed stable CP, especially those with significant growth failure and metabolic disturbances, as specified in the childhood cancer survivors (CCS) Consensus Statement (14). For children who have stable disease and concomitant GH deficiency, it is recommended to consider rGH therapy, which serves not only to improve adult height but also offers valuable metabolic benefits (15). Furthermore, it is cautioned against substituting ACTH deficiency with prednisolone in children due to the well-documented growth impairment associated with it (16). Additionally, the diagnosis of central hypothyroidism depends on low free thyroxine levels combined with inappropriately normal or low levels of thyroid-stimulating hormone (TSH), rather than relying solely on low TSH levels (17).

Traditionally, treatment strategies for CP have favored a radical approach involving gross-total resection, aiming to cure CP patients. We noted that none of the CPs recurred after gross total resection, compared to about one-fifth of patients following subtotal resection (2). However, evolving insights into risk-adapted therapy regimens have emerged over time, particularly for unfavorably located CP with hypothalamic involvement (HI) (18). The hypothalamic syndrome encompassing sleep-wake cycle disorders, temperature dysregulation, behavioral problems, and hypothalamic obesity (HO), poses significant challenges in the treatment strategy of CP patients (19). Namely, HO associated with impaired QoL are frequent and severe disabilities among CP patients (19). Therefore, strategies that spare the hypothalamus are highly recommended (2, 11, 20, 21), balancing radical resection for maximum safety with adjuvant treatment to control the disease in pediatric CP cases (22). Consequently, outcome parameters such as assessing HO are of paramount importance. Miao et al. (1) observed obesity in 54% of patients with recorded follow-up, while at baseline, it was noted in 76 out of 200 patients (38%). It can be assumed that weight gain was a substantial issue in this patient cohort. However, in CO cases, age-specific changes in weight gain and metabolic parameters should also be considered (23, 24), as they have been reported to significantly change during long-term follow-ups (2).

Regarding the comprehensive assessment of hypothalamic-pituitary dysfunction, evaluating treatment-related lesions of the optic apparatus and endocrine deficiencies only partially captures the therapeutic challenges in CP patients. This condition, especially in CO cases, is a chronic, incurable disease with anticipated lifelong effects, necessitating ongoing and multidisciplinary care for proper management of clinical and neuropsychological sequelae post-surgery. The assertion by the authors that ensuring adequate pituitary hormone replacement therapy and increasing engagement in sports activities are effective in managing postoperative obesity (25) is an overstatement. Namely, regarding the treatment of HO in CO CP patients, the results are ambiguous. While pharmacotherapies (26, 27) and bariatric procedures (28) demonstrate varying efficacy (29), none have been proven effective in randomized controlled trials (30). Thus, the impact of HO on quality of life and neuropsychological health, including social functioning during education and independent living, is considerable for long-term survivors (11, 31, 32). Therefore, it is advisable to prevent HO throughout hypothalamus-sparing surgical and radio-oncological strategies (21).

In summary, the treatment strategies for CO CP patients involve various complexities. Long-term follow-up studies are crucial to validate therapy effectiveness and ensure safety in managing this chronic disease. Strategies sparing the hypothalamus are advised to balance radical resection with the need for effective disease control. The impact of HO on patients’ quality of life is substantial, necessitating ongoing multidisciplinary care post-surgery. However, treatments for HO, such as pharmacotherapies and bariatric procedures, exhibit diverse effectiveness, emphasizing the need for further exploration in improving the well-being of long-term CP survivors.

## Author contributions

LA: Conceptualization, Writing – original draft, Writing – review & editing. EC: Conceptualization, Writing – review & editing.
